# Development and Research of a Theoretical Model of the Magnetic Tunnel Junction

**DOI:** 10.3390/s21062118

**Published:** 2021-03-17

**Authors:** Oleg Polyakov, Vladimir Amelichev, Dmitry Zhukov, Dmitry Vasilyev, Sergey Kasatkin, Peter Polyakov, Dmitry Kostyuk

**Affiliations:** 1 Faculty of Physics, Lomonosov Moscow State University, 119991 Moscow, Russia; physster@gmail.com (O.P.); polyakovpa@mail.ru (P.P.); 2 Scientific-Manufacturing Complex “Technological Centre”, 124498 Moscow, Russia; V.Amelichev@tcen.ru (V.A.); D.Vasilyev@tcen.ru (D.V.); D.Kostyuk@tcen.ru (D.K.); 3 V. A. Trapeznikov Institute of Control Sciences of RAS, 117997 Moscow, Russia; kasatkin14@mail.ru

**Keywords:** magnetic tunnel junction, theory of micromagnetism, magnetoresistive effect

## Abstract

Spin-dependent tunneling structures are widely used in many spintronic devices and sensors. This paper describes the magnetic tunnel junction (MTJ) characteristics caused by the inhomogeneous magnetic field of ferromagnetic layers. The extremely oblate magnetic ellipsoids have been used to mimic these layers. The strong effect of an inhomogeneous magnetic field on the magnetoresistive layers’ interaction was demonstrated. The magnetostatic coupling coefficient is also calculated.

## 1. Introduction

Spin-tunnel magnetoresistive nanostructures are used in various spintronic devices: in magnetic field sensors [1,2,3,4], in magnetoresistive biosensors [5], and in magnetoresistive memory elements [6,7,8]. The magnetic tunnel junction (MTJ) consists of a conducting free magnetic layer (FL), a dielectric tunnel barrier, and a conducting fixed magnetic layer (FixL) [7,8]. The vector of magnetization ${\vec{M}}_{2}$ in the FL has two equilibrium stable states. It is determined by the direction of the anisotropy axis, and can be transferred from one state to another by the magnetic field of electric current pulses in planar conductors. In this paper, we study the model of the MTJ and the process of remagnetization of its state by an external magnetic field. The magnetization vector ${\vec{M}}_{1}$ of the FixL is directed along the anisotropy axis. It has a high magnetization reversal field and does not change its direction when the orientation of ${\vec{M}}_{2}$ in the FL is changed. 

In some cases of practical use (the element of the magnetoresistive memory cell, the magnetosensitive element of a magnetically controlled scheme, the threshold sensor, etc.), it is necessary to have two stable states of the magnetization vector of the FL, which are defined through a change in the magnetoresistance of the MTJ. For example, for the stable operation of an MTJ-based bistable element, it is necessary to find its optimal geometric parameters and values of the magnetic field vector at which its state is switched. Research in this direction for various modifications of MTJ has been actively carried out for over 20 years [7,8]. For example, in [9], a theoretical study of the range of magnetic fields in which the direction of the vector $\vec{M}$ in the FL switches from one stable state to another was carried out. Magnetic stripes were modeled as strongly flattened identical ellipsoids with an equal magnetization. The density of the magnetostatic energy of the interaction of the stripes is considered to be

(1) $$w_{c}=-\left({\vec{M}}_{2}{\vec{H}}_{m1}\right)$$

where ${\vec{M}}_{2}$ is the magnetization vector of the second stripe; and ${\vec{H}}_{m1}$ is the magnetic field created by the first stripe in the area of the second stripe, which was considered uniform and coinciding with the demagnetizing field inside the ellipsoid—that is, inside the first stripe. The same author in [4] developed the theory for the case of stripes with different thicknesses (ellipsoids). In [5], an attempt was made to take into account the difference between the magnetic field of the FixL outside the stripe and the demagnetization field inside this stripe, assuming that this field is proportional to the demagnetization field. It was made by involving a certain decreasing constant coefficient, *r* = 0.8, which is assumed to be

(2) $${\vec{H}}_{m2}=r{\vec{H}}_{m1}$$

In this research, we study the range of stable operation of the MTJ by analogy with the research in [3,4,5], using the exact expression for the magnetic field of a uniformly magnetized ellipsoid.

## 2. Main Provisions of the Theory

**Figure 1:**
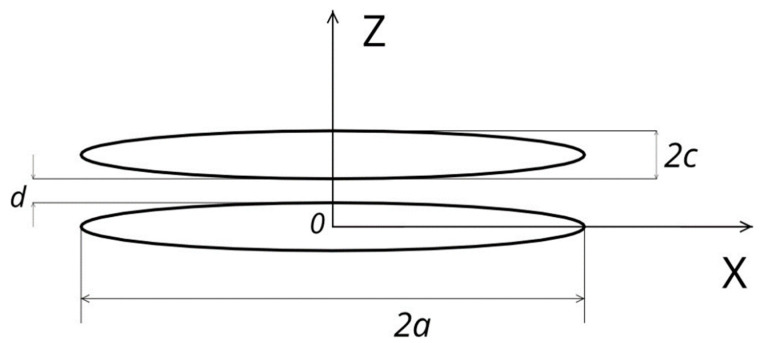
Section of the ellipsoidal magnetic stripes of the magnetic tunnel junction (MTJ) along the X*0*Z coordinate plane.

In accordance with [9,10,11,12,13,14,15], we consider two ferromagnetic layers in the form of uniformly magnetized ellipsoids strongly flattened along the Z coordinate axis (Figure 1). The semi-axes of ellipsoids a>b>c are oriented along the Cartesian axes of the X, Y, and Z coordinates. Figure 1 shows the cross section of these ellipsoids along the X*0*Z coordinate plane.

The lower ellipsoidal magnetic stripe is magnetized along the X coordinate axis and is hard magnetic with an infinitely large coercive force (FixL of the MTJ); that is, the magnetization vector of this stripe ${\vec{M}}_{1}$ is always oriented along the X coordinate axis. The upper ellipsoidal magnetic stripe (FL of the MTJ) has a crystal anisotropy and shape anisotropy along the X coordinate axis. Accordingly, it has two stable orientations of the magnetization vector ${\vec{M}}_{2}$ of this layer—along and against the X coordinate axis. The geometric dimensions of the layers are shown in Figure 1, where *d* is the distance between the layers; i.e., the thickness of the dielectric tunnel barrier of the MTJ. In general, the thicknesses *c* of the ferromagnetic ellipsoids (films) may not coincide.

The magnetostatic field outside the ellipsoid will be inhomogeneous and can only be considered approximately equal to the homogeneous demagnetizing field inside the FixL; this was assumed in [9,10,11,12,13,14]. To calculate this field, we use the well-known expression for the magnetostatic potential of a uniformly magnetized ellipsoid outside this ellipsoid [12]:

(3) $$\varphi (x,y,z)=4\pi \frac{abc}{2}\int_{\xi (x,y,z)}^{\infty }\frac{dt}{\left(a^{2}+t\right)R(t)}\cdot M_{1x}x$$

where

(4) $$R\left(t\right)=\sqrt{\left(a^{2}+t\right)\left(b^{2}+t\right)\left(c^{2}+t\right)}$$

It is taken into account that the magnetization vector ${\vec{M}}_{1}$ is directed along the X axis and has the only non-zero component:

(5) $$M_{1x}=\left|{\vec{M}}_{1}\right|=M$$

The value of ξ is an ellipsoidal coordinate, depending on Cartesian coordinates ξ(x,y,z) that are determined by solving the following equation [13]:

(6) $$\frac{x^{2}}{a^{2}+\xi }+\frac{y^{2}}{b^{2}+\xi }+\frac{z^{2}}{c^{2}+\xi }=1$$

The projection of the magnetic field strength vector $H_{x}$ outside the ellipsoid according to Expression (3) will be equal to

(7) $$H_{x}=-\frac{\partial \varphi }{\partial x}=4\pi \frac{abc}{2}\left(\frac{x}{\left(a^{2}+\xi \right)R(\xi )}\frac{\partial \xi (x,y,z)}{\partial x}-\int_{\xi (x,y,z)}^{\infty }\frac{dt}{\left(a^{2}+t\right)R(t)}\right)\cdot M_{1x}$$

Equation (7) is expressed in terms of the incomplete elliptic integrals of the first and second kind. The ξ(x,y,z) can be simplified at *y* = 0 and, according to (6), will be determined by the following expression:

(8) $$\xi \left(x,y,z\right)=\sqrt{{\left(\frac{a^{2}+c^{2}-x^{2}-z^{2}}{2}\right)}^{2}-a^{2}c^{2}+x^{2}c^{2}+z^{2}a^{2}}-\left(\frac{a^{2}+c^{2}-x^{2}-z^{2}}{2}\right)$$

**Figure 2:**
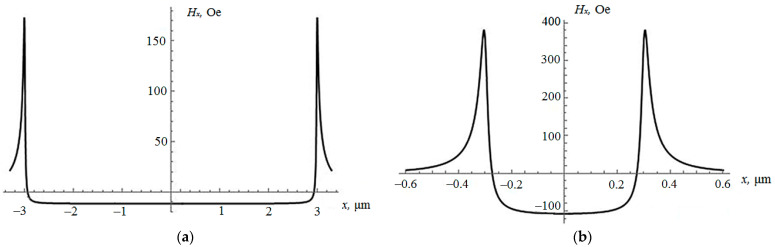
Dependence of the strength projection $H_{x}\;$on the X coordinate along the semi-major axis of the upper ellipsoid: (**a**) for *a* = 3 μm, *b* = 1 μm, *c* = 5 nm, and *d* = 3 nm; (**b**) for *a* = 0.3 μm, *b* = 0.1 μm, *c* = 5 nm, and *d* = 3 nm.

Formulas (7) and (8) were used to calculate the dependence of the $H_{x}$ strength projection on the X coordinate along the semi-major axis of the upper ellipsoid, where z=2c+d (Figure 1), for magnetic stripes made of FeNiCo with a saturation magnetic moment of *M* = 1050 G, for two cases: with *a* = 3 μm, *b* = 1 μm, *c* = 5 nm, and *d* = 3 nm; and with *a* = 0.3 μm, *b* = 0.1 μm, *c* = 5 nm, and *d* = 3 nm. Graphs of this dependence are shown in Figure 2.

From Figure 2, it should be concluded that, in the first case, the projection of the strength of the magnetic field along the major axis of symmetry of the ellipsoid is basically almost constant and close to the value in the center of the ellipsoid $H_{x}\left(0\right)=-11.57$ Oe. The value of the demagnetizing field inside the lower ellipsoidal stripe is $H_{m1x}$ = −11.63 Oe. So, on most of the upper ellipsoid, the magnetic field generated by the lower FixL stripe coincides with an accuracy of 0.1%, which proves the approximation of the study [9]. However, near the ends of the semi-axis of the upper ellipsoid in an area of the order of 0.1*a*, the field sharply changes its sign and reaches the value at the apex of the ellipsoid $H_{x}\left(a\right)=152.77$ Oe. The average value along the major axis of the upper ellipsoid calculated by Formula (7) is $H_{X\mathrm{cp}}=-10.23\;$Oe. If we formally calculate the magnetostatic coupling parameter *r* in (2), introduced in [11], by the formula

(9) $$r=\frac{H_{xav}}{H_{m1}}$$

then, for (9), we get the value *r* = 0.88.

In the second case, when the length and width of the stripes are ten times less (*a* = 0.3 μm, *b* = 0.1 μm), from Figure 2b, it can be seen that the inhomogeneity of the field inside the upper ellipsoid increases significantly. The demagnetizing field inside the lower ellipsoid is$\;H_{m1x}=-113.13$ Oe. The field in the center of the upper ellipsoid generated by the magnetized lower ellipsoid is $H_{x}\left(0\right)=-107.29$ Oe and differs by 5% from$\;H_{m1x}$; at the top of the ellipsoid, the value is $H_{x}\left(a\right)=350.04$ Oe. The average value along the larger axis of the upper ellipsoid is $H_{xav}=-73.92$ Oe. The parameter *r* in (9) will have the following value: *r* = 0.65. 

From the above data, it follows that if the FL and FixL of the MTJ are identical, then at a depth of about 10% from the top of the FL (the large poles of the upper ellipsoid in Figure 1) there is a strong inhomogeneity in the magnetization distribution, which may affect the resistance of the MTJ. To get rid of this feature, it is enough to reduce the FL in size by about of 10%, then the sensitive layer will be located in a more uniform and significantly smaller magnetostatic coupling field.

## 3. Range of Bistable Quasi-Equilibrium States of FL

The range of a bistable quasi-equilibrium state of a uniformly magnetized ellipsoidal particle in an external magnetic field has been studied for various specific physical problems [7,9,10,11]. These similar studies took into account the inhomogeneity of the magnetic field of FixL (the field created by the magnetization vector of the lower ellipsoid in Figure 1). We assume that the distribution of the magnetization vectors in the stripes is uniform. Then, the magnetic energy density of the FL (in the upper ellipsoid in Figure 1) can be represented as

(10) $$w=w_{z}+w_{an}+w_{m}+w_{c}$$

where $w_{z}$ is the magnetic energy density caused by an external field ${\vec{H}}_{0}$ (Zeeman energy), then

(11) $$w_{z}=-{\vec{M}}_{2}{\vec{H}}_{0}$$

where $w_{an}$ is the monoaxial anisotropy magnetic energy density:

(12) $$w_{an}=K\cdot \sin^{2}\varphi$$

where *K* is a constant of the mono-axial anisotropy whose axis is directed along the X coordinate axis; and φ is the angle between the magnetization vector ${\vec{M}}_{2}$ and the direction of the X coordinate axis. Expression (12) is often written using the constant “anisotropy field”.

(13) $$H_{an}=\frac{2K}{M}$$

where

(14) $$M=\left|{\vec{M}}_{1}\right|=\left|{\vec{M}}_{2}\right|$$

Taking into account (13), Formula (12) transforms into

(15) $$M=\left|{\vec{M}}_{1}\right|=\left|{\vec{M}}_{2}\right|$$

The third term in (10), $w_{m}$, is the density of magnetostatic energy,

(16) $$w_{m}=-\frac{{\vec{H}}_{m}\cdot {\vec{M}}_{2}}{2}$$

where ${\vec{H}}_{m}$ is the demagnetizing field of an ellipsoid whose components are defined by the following expression:

(17) $$H_{mx}=-4\pi n_{x}M_{2x},\;\;\;H_{my}=-4\pi n_{y}M_{2y},$$

$n_{x}\;$and $n_{y}$ are the demagnetizing coefficients of the ellipsoid [13]:

(18) $$n_{x}=\frac{abc}{2}\int_{0}^{\infty }\frac{dt}{\left(a^{2}+t\right)R(t)},\;\;\;n_{y}=\frac{abc}{2}\int_{0}^{\infty }\frac{dt}{\left(b^{2}+t\right)R(t)}$$

The last term in (10) is the density of the magnetostatic energy of the interaction with the magnetostatic field of the FixL (the magnetic field of the lower ellipsoid in Figure 1). In contrast to [9,10,11,14], we took into account the influence of the inhomogeneity of this field. For a simpler calculation, this energy was taken near the larger axis of symmetry of the ellipsoid; that is, in a narrow cylinder of radius ε and height 2*a*, within which the field can be considered to coincide with the field on the axis of symmetry, having one non-zero component $H_{x}$, which is (7). Then, the energy of interaction with this area $W_{c}$ will be equal to

(19) $$W_{c}=\int w_{c}dV=-\int_{-a}^{a}M_{2x}H_{x}dx\cdot \pi \varepsilon^{2}$$

As $M_{2x}$ is constant, it can be taken outside the integral, so Expression (19) becomes the following:

(20) $$W_{c}=-M_{2x}\frac{1}{2a}\int_{-a}^{a}H_{x}dx\cdot \pi \varepsilon^{2}\cdot 2a$$

Taking into account that the integral in (20) is equal to the mean value of the field $H_{x}$ along the axis of the ellipsoid,

(21) $$H_{xav}=\frac{1}{2a}\int_{-a}^{a}H_{x}dx$$

From (20), for the interaction energy density, we get the following:

(22) $$w_{c}=\frac{W_{c}}{\pi \varepsilon^{2}2a}=-M_{2x}H_{xav}$$

Taking into account the value of the components of the vectors $\vec{M_{2\;}}$ and $\vec{H_{0}}$

(23) $$\;M_{2x}=M\cos\varphi ,\;M_{2y}=M\sin\varphi ,$$

(24) $${\vec{H}}_{0}=\left(H_{0x},H_{0y},0\right)$$

From Formulas (13)–(22), we obtain the following dependence of the magnetic energy density (10) on the angle of rotation φ of the magnetization vector ${\vec{M}}_{2}$ to the X coordinate axis:

(25) $$\begin{array}{r} w\left(\varphi \right)=-H_{0x}M\cos\varphi -H_{0y}M\sin\varphi +\frac{1}{2}H_{an}M\sin^{2}\varphi +\\ +2\pi \left(n_{x}M^{2}\cos^{2}\varphi +n_{y}M^{2}\sin^{2}\varphi \right)-H_{xav}M\cos\varphi \end{array}$$

Expression (25) can easily be transformed to

(26) $$w\left(\varphi \right)=M\left[-\left(H_{0x}+H_{xav}\right)\cos\varphi -H_{0y}\sin\varphi +\frac{H_{an}+4\pi n_{y}M}{2}\sin^{2}\varphi +2\pi n_{x}M\cos^{2}\varphi \right]$$

Energy density (26), depending on the values of the components of the external field $\;H_{0x}$,$\;H_{0y}$ and the average magnetostatic field $H_{xav}$, can have two local minimums (bistable state) or one minimum (one stable state of vector ${\vec{M}}_{1}$ orientation). As the external magnetic field components increase$\;H_{0x}$,$\;H_{0y}$, while the angle φ is fixed, the bistable state changes into one stable state. Mathematically, it means that the function has three extreme points (26): two local minimums and a maximum. The critical values of the parameters $\;H_{0x}$,$\;H_{0y}$, which make a bistable state change, mean that one local minimum merges a local maximum in the function (26) and the forming of an inflection point. The values of $\;H_{0x}$,$\;H_{0y}$ at which such a state occurs can be determined using the following [14]:

(27) $$\frac{\partial w\left(\varphi \right)}{\partial \varphi }=0,\;\;\;\;\frac{\partial^{2}w\left(\varphi \right)}{\partial^{2}\varphi }=0$$

After differentiating Function (26), we get the following:

(28) $$\left(H_{0x}+H_{xav}\right)\sin\varphi -H_{0y}\cos\varphi +\frac{H_{an}+4\pi \left(n_{y}-n_{x}\right)M}{2}\sin2\varphi =0,$$

(29) $$\left(H_{0x}+H_{xav}\right)\cos\varphi +H_{0y}\sin\varphi +\left(H_{an}+4\pi \left(n_{y}-n_{x}\right)M\right)\cos2\varphi =0$$

The system of Equations (28) and (29) is easy to convert to the following equivalent system of equations:

(30) $$H_{0x}+H_{xav}=-\left(H_{an}+4\pi M\left(n_{y}-n_{x}\right)\right)\cos^{3}\varphi$$

(31) $$H_{0y}=\left(H_{an}+4\pi M\left(n_{y}-n_{x}\right)\right)\sin^{3}\varphi$$

While the angle φ changes from 0 to 2π, Equations (30) and (31) will draw on the plane a closed astroid curve, the inversion curve, which was first obtained by Stoner and Wohlfarth [7,14].

**Figure 3:**
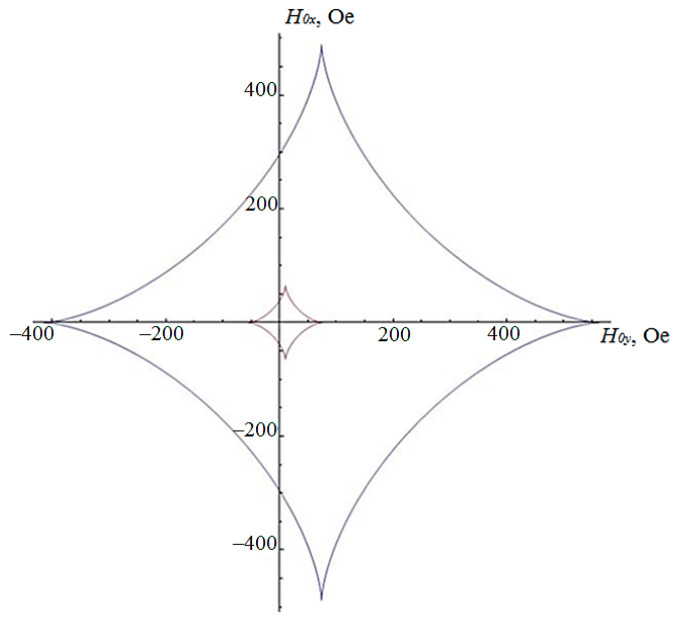
Stoner–Wohlfarth curves for two free magnetic layers (FLs) of the MTJ with ellipsoid semi-axes of *a* = 3 μm and *b* = 1 μm (inner curve); and *a* = 0.3 μm and *b* = 0.1 μm (outer curve).

The Stoner–Wohlfarth curves, constructed by Formulas (30) and (31), for the two FLs of the MTJ considered in paragraph 2, are shown in Figure 3. The inner astroid in Figure 3 corresponds to the sample with the semi-major axis of the ellipsoid *a* = 3 μm and *b* = 1 μm, and the outer astroid with the minor semi-axis *a* = 0.3 μm and *b* = 0.1 μm. The difference between this curve and the classical astroid [7,13] is due to its shift along the X axis by the mean field value (21).

For the values of the external field components $H_{0x}$,$\;H_{0y}$ equal to the inner points of the astroid in Figure 3 there is a bistable state, i.e., two stable orientations of the magnetization vector $\;{\vec{M}}_{2}$, and outside of the astroid, only one equilibrium state of ${\vec{M}}_{2}$ is possible [7,13,14]. Thus, in order to transfer the MTJ from a low-resistive to a highly-resistive state, or vice versa, it is necessary to act with a magnetic field, the components of which lie outside the critical Stoner–Wohlfarth curve. From Figure 3, it can be clearly seen that magnetization of the MTJ with a length and width an order of magnitude smaller, but with the same thickness, requires a magnetic field that is ten times larger. This occurs due to an increase in the demagnetizing coefficients *n_x_*, *n_y_* in (30) and (31) when reducing the semi-axes of the ellipsoids *a* and *b* by 10 times. The coefficients *n_x_*, *n_y_* can be reduced by decreasing the thickness of the magnetic layers; this may weaken the value of the tunnel magnetoresistive effect.

Let us now determine what is the minimum pulsed magnetic field *H*_0_ needed to switch the MRAM cells. To do this, we write (30) and (31) in dimensionless form, introducing dimensionless values:

(32) $${\bar{H}}_{0x}=\frac{H_{0x}}{H_{an}+4\pi M\left(n_{y}-n_{x}\right)},\;{\bar{H}}_{0y}=\frac{H_{0y}}{H_{an}+4\pi M\left(n_{y}-n_{x}\right)},$$

(33) $${\bar{H}}_{xav}=\frac{H_{xav}}{H_{an}+4\pi M\left(n_{y}-n_{x}\right)}$$

Then, instead of (30) and (31), we get the following:

(34) $${\bar{H}}_{0x}=-{\bar{H}}_{xav}-\cos^{3}\varphi$$

(35) $${\bar{H}}_{0y}=\sin^{3}\varphi$$

The modulus of the dimensionless vector of the external field strength according to (34) and (35) will be

(36) $${\bar{H}}_{0}=\sqrt{{\bar{H}}_{0x}{}^{2}+{\bar{H}}_{0y}{}^{2}}=\sqrt{{\left({\bar{H}}_{xav}+\cos^{3}\varphi \right)}^{2}+\sin^{6}\varphi }$$

Differentiating the expression (36) for φ and equating the derivative to zero we get the equation for determining the value of the angle φ, at which ${\bar{H}}_{0}$ reaches its extreme points:

(37) $$2\cos^{2}\varphi +{\bar{H}}_{xav}\cos\varphi -1=0$$

From (37), we find two different values of cosφ, where the distance to different sections of the inversion curve in Figure 3 will be minimal:

(38) $$\cos\varphi_{1,2}=\frac{-{\bar{H}}_{xav}\pm \sqrt{{\left({\bar{H}}_{xav}\right)}^{2}+8}}{4}$$

For the MTJ described above, ${\bar{H}}_{xav}$= −0.1577 (for *a* = 3 μm and *b* = 1 μm) and ${\bar{H}}_{xav}$= −0.1513 (for *a* = 0.3 μm and *b* = 0.1 μm); so, they are almost equal.

(39) $$\cos\varphi_{1}=0.7476,\;\;\;\;\;\;\;\varphi_{1}=41.6{}^{\circ}$$

(40) $$\cos\varphi_{2}=-0.6687,\;\;\;\;\;\varphi_{2}=131.9{}^{\circ}$$

Taking (39) and (40) into expressions (34)–(36), we find

(41) $${\bar{H}}_{0x1}\;=-0.260,\;\;\;\;\;{\bar{H}}_{0y1}\;=0.292,\;\;\;{\bar{H}}_{01}\;=0.414$$

(42) $${\bar{H}}_{0x2}\;=0.457,\;\;\;\;\;{\bar{H}}_{0y2}\;=0.411,\;\;\;{\bar{H}}_{02}\;=0.614$$

According to the notation (32), multiplying (41) and (42) by the multiplier $H_{an}+4\pi M\left(n_{y}-n_{x}\right)$, receiving for the two FL stripes under consideration (with *a* = 3 μm and *b* = 1 μm and *a* = 0.3 μm and *b* = 0.1 μm) the values of 64.9 Oe and 488.6 Oe, we obtain the following optimal physical values of the field strength components that switch the orientation of the vector ${\vec{M}}_{2}$ in the FL. The calculation results are presented in Table 1 and Table 2.

## 4. Conclusions

The influence of the inhomogeneity of the magnetic field in the FixL of the MTJ on switching the orientations of the vector ${\vec{M}}_{2}$ in the FL was studied. It was found that near the boundaries of the magnetic stripe of the FL, the inhomogeneity of the magnetic field, caused by the magnetization of the FixL, reaches a significant value, and the magnetic field at the border increases sharply compared to the central part of the stripe. When the size of the MTJ decreases, the inhomogeneity of the magnetic field increases in the volume of the entire nanostructure. It is shown that if the distribution of the magnetization vector over the volume of the MTJ is uniform, this inhomogeneity can be taken into account by introducing the average field. This justifies the model developed in [11] and allows us to calculate the empirical coefficient introduced in this paper. Within the framework of the developed theory, the region of the stable equilibrium state of the vector in FL is calculated, and the critical Stoner–Wohlfarth curve is obtained. The optimal values of the magnetic field that switch the state of the MTJ are found.

## Figures and Tables

**Table 1:** For the first stripe with *a* = 3 μm and *b* = 1 μm.

*N*	$H_{0x},\;\mathrm{Oe}$	$H_{0y},\mathrm{Oe}$	$H_{0},\mathrm{Oe}$
1	−17	19	25
2	30	27	40

**Table 2:** For the second stripe with *a* = 0.3 μm and *b* = 0.1 μm.

*N*	$H_{0x},\;\mathrm{Oe}$	$H_{0y},\;\mathrm{Oe}$	$H_{0},\;\mathrm{Oe}$
1	−129	144	194
2	221	200	298
